# Supplementary material for: Behavioural variability among captive African elephants in the use of the trunk while feeding
Source: PeerJ. 2020 Aug 18;8:e9678. doi: 10.7717/peerj.9678 (PMC7441921; doi:10.7717/peerj.9678)
Supplement: Supplemental Information 1 [file peerj-08-9678-s001.docx]

Details of the PCA

Code for the behaviours

| a1: Pinch |
| --- |
| a2: Side pinch |
| a4: Grasp |
| a5: Torsion |
| a6: Bundling |
| a7: Shake |
| a8: Sweep |
| a9: Gather |
| a10: Pull |
| a15: Adjust |
| a18: Bring to mouth |
| a19: Block |

Code for the food items

| b1: branch ; Ø< 0.5 cm |
| --- |
| b2: branch ; 1 < Ø < 2 cm |
| b3: branch ; Ø > 2 cm |
| h: hay |
| v: vegetables |
| a: apple |

Table of data

| Individual | Food item | a1 | a2 | a4 | a5 | a6 | a7 | a8 | a9 | a10 | a15 | a18 | a19 |
| --- | --- | --- | --- | --- | --- | --- | --- | --- | --- | --- | --- | --- | --- |
| A1 | b1 | 30.5 | 0.0 | 17.3 | 6.7 | 6.5 | 0.5 | 0.0 | 0.0 | 11.1 | 0.0 | 27.4 | 0.0 |
| A2 | b1 | 29.5 | 0.0 | 18.6 | 16.8 | 4.1 | 0.0 | 0.0 | 0.0 | 2.4 | 0.7 | 27.9 | 0.0 |
| A3 | b1 | 30.1 | 0.0 | 22.6 | 10.6 | 2.1 | 0.0 | 0.0 | 0.0 | 4.1 | 2.1 | 28.4 | 0.0 |
| A4 | b1 | 25.5 | 0.0 | 24.4 | 16.2 | 4.8 | 0.0 | 0.0 | 0.0 | 5.9 | 1.1 | 21.4 | 0.7 |
| B1 | b1 | 28.5 | 0.0 | 22.2 | 7.5 | 2.8 | 0.2 | 0.0 | 0.0 | 13.2 | 0.5 | 25.0 | 0.0 |
| B2 | b1 | 25.7 | 0.0 | 21.5 | 21.3 | 6.2 | 0.4 | 0.7 | 0.0 | 0.4 | 0.2 | 23.5 | 0.0 |
| A1 | b2 | 29.9 | 0.0 | 22.3 | 9.6 | 2.5 | 0.6 | 0.0 | 0.0 | 4.5 | 7.6 | 22.9 | 0.0 |
| A2 | b2 | 28.5 | 0.0 | 19.7 | 12.4 | 3.6 | 0.0 | 0.0 | 0.0 | 2.9 | 8.0 | 24.8 | 0.0 |
| A3 | b2 | 24.8 | 0.0 | 25.7 | 6.9 | 1.0 | 0.0 | 0.0 | 0.0 | 9.9 | 8.9 | 22.8 | 0.0 |
| A4 | b2 | 23.3 | 0.0 | 29.3 | 21.8 | 3.0 | 0.0 | 0.0 | 0.0 | 1.5 | 3.8 | 14.3 | 3.0 |
| B1 | b2 | 30.3 | 0.0 | 25.1 | 15.2 | 2.4 | 0.0 | 0.0 | 0.0 | 4.7 | 1.9 | 20.4 | 0.0 |
| B2 | b2 | 26.6 | 0.0 | 23.8 | 21.1 | 3.1 | 0.0 | 0.0 | 0.0 | 0.4 | 3.1 | 20.7 | 1.2 |
| A1 | b3 | 26.2 | 0.0 | 26.2 | 13.8 | 0.0 | 0.0 | 0.0 | 0.0 | 0.0 | 10.8 | 23.1 | 0.0 |
| A2 | b3 | 22.8 | 0.0 | 21.1 | 1.6 | 0.0 | 0.0 | 0.0 | 0.0 | 1.6 | 32.5 | 19.5 | 0.8 |
| A3 | b3 | 25.0 | 0.0 | 28.3 | 5.0 | 0.0 | 0.0 | 0.0 | 0.0 | 10.0 | 11.7 | 18.3 | 1.7 |
| A4 | b3 | 29.4 | 0.0 | 22.4 | 3.5 | 0.0 | 0.0 | 0.0 | 0.0 | 1.2 | 24.7 | 17.6 | 1.2 |
| B1 | b3 | 40.0 | 0.0 | 17.8 | 0.0 | 5.6 | 0.0 | 0.0 | 0.0 | 10.0 | 11.1 | 15.6 | 0.0 |
| B2 | b3 | 36.3 | 0.0 | 17.7 | 12.4 | 6.2 | 0.0 | 0.0 | 0.0 | 0.9 | 5.3 | 17.7 | 3.5 |
| A1 | h | 40.6 | 0.2 | 2.5 | 0.0 | 23.2 | 5.8 | 0.0 | 0.9 | 0.1 | 0.1 | 26.4 | 0.1 |
| A2 | h | 26.8 | 0.0 | 15.1 | 0.0 | 23.2 | 9.0 | 1.1 | 0.9 | 0.4 | 0.0 | 23.4 | 0.1 |
| A3 | h | 35.1 | 0.0 | 4.9 | 0.0 | 19.8 | 17.1 | 0.0 | 0.6 | 0.1 | 0.1 | 22.0 | 0.4 |
| A4 | h | 50.5 | 0.0 | 9.9 | 0.0 | 20.6 | 5.6 | 0.2 | 0.6 | 0.2 | 0.0 | 12.5 | 0.0 |
| B1 | h | 25.8 | 0.0 | 20.5 | 0.0 | 16.6 | 19.0 | 1.6 | 0.9 | 0.0 | 0.0 | 15.2 | 0.2 |
| B2 | h | 32.2 | 0.0 | 1.6 | 0.1 | 24.9 | 21.5 | 0.3 | 0.8 | 0.4 | 0.0 | 18.0 | 0.1 |
| A1 | v | 44.7 | 2.5 | 0.0 | 0.0 | 9.4 | 0.0 | 0.0 | 1.9 | 0.0 | 0.0 | 41.5 | 0.0 |
| A2 | v | 39.0 | 3.5 | 0.0 | 0.0 | 5.0 | 0.0 | 3.5 | 0.0 | 0.0 | 0.0 | 48.9 | 0.0 |
| A3 | v | 37.2 | 17.3 | 0.0 | 0.0 | 3.2 | 0.0 | 0.0 | 1.9 | 0.0 | 0.0 | 40.4 | 0.0 |
| A4 | v | 53.1 | 1.6 | 0.0 | 0.0 | 10.9 | 0.0 | 0.0 | 0.8 | 0.0 | 0.0 | 33.6 | 0.0 |
| B1 | v | 19.0 | 27.0 | 4.0 | 0.0 | 5.0 | 0.0 | 3.0 | 3.0 | 0.0 | 0.0 | 39.0 | 0.0 |
| B2 | v | 42.0 | 0.5 | 0.0 | 0.0 | 17.5 | 0.0 | 0.0 | 0.0 | 0.0 | 0.0 | 40.0 | 0.0 |
| A1 | a | 41.6 | 9.0 | 0.0 | 0.0 | 0.5 | 0.0 | 0.0 | 6.3 | 0.0 | 0.0 | 42.5 | 0.0 |
| A2 | a | 52.3 | 2.0 | 0.0 | 0.0 | 0.0 | 0.0 | 0.0 | 1.3 | 0.0 | 0.0 | 44.4 | 0.0 |
| A3 | a | 61.5 | 0.0 | 0.0 | 0.0 | 0.0 | 0.0 | 0.0 | 0.0 | 0.0 | 0.0 | 38.5 | 0.0 |
| A4 | a | 31.3 | 20.7 | 0.0 | 0.0 | 1.4 | 0.0 | 1.8 | 5.1 | 0.0 | 0.0 | 39.6 | 0.0 |
| B1 | a | 13.3 | 38.3 | 0.0 | 0.0 | 1.7 | 0.0 | 1.7 | 6.7 | 0.0 | 0.0 | 38.3 | 0.0 |
| B2 | a | 35.4 | 13.4 | 0.0 | 0.0 | 0.0 | 0.0 | 0.0 | 4.9 | 0.0 | 0.0 | 46.3 | 0.0 |

Results from the normed PCA

| Component | Eigen value | % Total |
| --- | --- | --- |
| 1 | 4.29 | 35.78 |
| 2 | 2.37 | 19.75 |
| 3 | 1.61 | 13.42 |
| 4 | 1.04 | 8.63 |
| 5 | 0.96 | 7.98 |
| 6 | 0.66 | 5.54 |
| 7 | 0.52 | 4.29 |
| 8 | 0.22 | 1.84 |
| 9 | 0.17 | 1.45 |
| 10 | 0.11 | 0.93 |
| 11 | 0.05 | 0.40 |

Contribution of the different variables to each component

| Behaviour | Comp. 1 | Comp. 2 | Comp. 3 | Comp. 4 | Comp. 5 | Comp. 6 | Comp. 7 | Comp. 8 | Comp. 9 | Comp.10 | Comp.11 |
| --- | --- | --- | --- | --- | --- | --- | --- | --- | --- | --- | --- |
| a1 | -0.348 | 0.441 | -0.763 | -0.177 | -0.010 | -0.063 | -0.129 | 0.061 | -0.215 | -0.010 | 0.020 |
| a2 | -0.676 | -0.552 | 0.365 | -0.003 | -0.042 | 0.185 | -0.038 | -0.143 | -0.092 | -0.198 | -0.021 |
| a4 | 0.933 | -0.171 | 0.193 | 0.124 | 0.066 | -0.032 | 0.067 | 0.055 | -0.087 | 0.082 | -0.143 |
| a5 | 0.707 | -0.267 | 0.069 | -0.164 | 0.576 | -0.005 | 0.225 | -0.002 | -0.002 | -0.013 | 0.118 |
| a6 | -0.184 | 0.877 | 0.296 | 0.026 | 0.054 | 0.040 | -0.039 | -0.299 | 0.062 | 0.094 | 0.010 |
| a7 | -0.107 | 0.776 | 0.508 | 0.067 | -0.091 | 0.121 | 0.057 | 0.294 | 0.053 | -0.092 | 0.021 |
| a8 | -0.507 | -0.197 | 0.540 | 0.100 | 0.071 | -0.580 | -0.207 | 0.052 | -0.101 | 0.064 | 0.038 |
| a9 | -0.744 | -0.383 | 0.209 | -0.072 | -0.079 | 0.429 | 0.049 | 0.088 | -0.080 | 0.207 | 0.037 |
| a10 | 0.544 | -0.183 | -0.144 | 0.674 | -0.032 | 0.181 | -0.395 | 0.009 | 0.038 | 0.002 | 0.062 |
| a15 | 0.532 | -0.247 | -0.002 | -0.124 | -0.762 | -0.158 | 0.165 | -0.039 | 0.039 | 0.015 | 0.068 |
| a18 | -0.807 | -0.332 | -0.341 | 0.006 | 0.123 | -0.144 | 0.002 | 0.068 | 0.282 | 0.027 | -0.033 |
| a19 | 0.509 | -0.097 | 0.186 | -0.686 | -0.004 | 0.100 | -0.457 | 0.028 | 0.075 | 0.003 | -0.007 |

Correlations between variables

|  | a1 | a2 | a4 | a5 | a6 | a7 | a8 | a9 | a10 | a15 | a18 | a19 |
| --- | --- | --- | --- | --- | --- | --- | --- | --- | --- | --- | --- | --- |
| a1 | 1.00 | -0.28 | -0.56 | -0.42 | 0.19 | -0.03 | -0.25 | -0.07 | -0.25 | -0.28 | 0.34 | -0.20 |
| a2 | -0.28 | 1.00 | -0.49 | -0.34 | -0.23 | -0.18 | 0.53 | 0.82 | -0.28 | -0.23 | 0.53 | -0.20 |
| a4 | -0.56 | -0.49 | 1.00 | 0.73 | -0.28 | -0.13 | -0.30 | -0.59 | 0.55 | 0.47 | -0.76 | 0.40 |
| a5 | -0.42 | -0.34 | 0.73 | 1.00 | -0.33 | -0.30 | -0.28 | -0.43 | 0.21 | 0.07 | -0.44 | 0.40 |
| a6 | 0.19 | -0.23 | -0.28 | -0.33 | 1.00 | 0.76 | 0.06 | -0.14 | -0.26 | -0.36 | -0.24 | -0.12 |
| a7 | -0.03 | -0.18 | -0.13 | -0.30 | 0.76 | 1.00 | 0.10 | -0.05 | -0.22 | -0.21 | -0.34 | -0.08 |
| a8 | -0.25 | 0.53 | -0.30 | -0.28 | 0.06 | 0.10 | 1.00 | 0.32 | -0.28 | -0.23 | 0.36 | -0.18 |
| a9 | -0.07 | 0.82 | -0.59 | -0.43 | -0.14 | -0.05 | 0.32 | 1.00 | -0.35 | -0.29 | 0.57 | -0.24 |
| a10 | -0.25 | -0.28 | 0.55 | 0.21 | -0.26 | -0.22 | -0.28 | -0.35 | 1.00 | 0.19 | -0.35 | 0.01 |
| a15 | -0.28 | -0.23 | 0.47 | 0.07 | -0.36 | -0.21 | -0.23 | -0.29 | 0.19 | 1.00 | -0.41 | 0.29 |
| a18 | 0.34 | 0.53 | -0.76 | -0.44 | -0.24 | -0.34 | 0.36 | 0.57 | -0.35 | -0.41 | 1.00 | -0.44 |
| a19 | -0.20 | -0.20 | 0.40 | 0.40 | -0.12 | -0.08 | -0.18 | -0.24 | 0.01 | 0.29 | -0.44 | 1.00 |

Bartlett sphericity test

Khi² = 716.08 ; ddl = 66, *P* < 0.
